# Chemical injuries from assaults: An increasing trend in a developing country

**DOI:** 10.4103/0970-0358.41106

**Published:** 2008

**Authors:** Peter B. Olaitan, Bernard C. Jiburum

**Affiliations:** Burns and Plastic Surgery Unit, Department of Surgery, Ladoke Akintola University of Technology Teaching Hospital, Osogbo, Osun State, Nigeria; 1 Imo state University Owerri, Imo state, Nigeria

**Keywords:** Assault, chemical injuries, Nigeria, prevention

## Abstract

**Objective:**

This paper describes chemical injuries, which presented to us and were managed at a burn unit in Nigeria. The purpose of this paper is to highlight the etiologies of these injuries, the extent of the injuries as well as to suggest possible ways to prevent chemical injuries in our environment.

**Materials and Methods:**

We carried out a retrospective review of chemical burns treated at our center. Our sources of information were the burn unit admission registers, case notes of the patients and operation registers. The results were collated and then analyzed.

**Results:**

Twenty eight patients presented with chemical burn injuries during the study period between January 2000 and December 2003, constituting 5.7% of all patients with burns treated within that period. Seventeen (60.7%) of the patients were males while 11 (29.3%) were females with a mean age of 20.6 years. The injuries were sustained from assault in 21 (75%), armed robbery attacks in five (17.8%) and suicide attempts in two (7.1%). The agents were usually unknown. Late presentation was observed in all the patients. Raw eggs, palm oil, gentian violet and engine oil were the substances applied immediately after the injuries. Complications observed included septicemia, respiratory distress, blindness, renal failure, mentosternal contractures, ectropion, axillary contractures, hypertrophic scars, keloids and skin depigmentation.

**Conclusion:**

Chemical burn injuries are mainly due to assaults in Nigeria and are usually extensive and presented late. Education of the people and penalty for any offender will reduce the current spate of such injuries.

## INTRODUCTION

Chemical burn injuries occur when certain substances come into direct contact with the skin or mucous membranes with the onset of rapid tissue damage. Chemical burn injuries are not uncommon and have been reported by various authors.[1–5]

Epidemiology of chemical burns varies not only from one country to another but also among institutions, depending on the geographic location, the surrounding industry and social environment (war or peace time).

The current series is from a tertiary center in Nigeria with a burn unit where burns’ cases are referred. These include extensive chemical burns with the patient presenting much later after the injuries when dilution of the chemical by water will not be too useful. We stress the peculiarity of these cases both in etiology and extent and suggest the means of preventing them.

## MATERIALS AND METHODS

The admission registers and clinical charts of all cases of chemical burns admitted between January 2000 and December 2003 were retrospectively reviewed. Data collected included age, sex, etiology, place and site of injury, total body surface area (TBSA) affected, types of injuries and presentation, methods of management, complications and prognosis. The data was collated and analyzed.

## RESULTS

Out of 485 patients who presented with burn injuries during the study period from January 2000 to December 2003, 28 (5.8%) were observed to suffer from chemical burn injuries.

Our center is a regional burns center established by the Federal Government of Nigeria to care for the people of Eastern Nigeria.

Seventeen (60.7%) of the patients with chemical injuries were males while 11 (29.3%) were females. Their ages ranged between 2-60 years with a mean of 20.6 years. The injuries were sustained from assault in 21 (75%), armed robbery attacks in five (17.8%) and suicide attempts in two (7.1%). The agents were unknown except in the cases of suicide where sulphuric acid was used.

**Figure 1:**
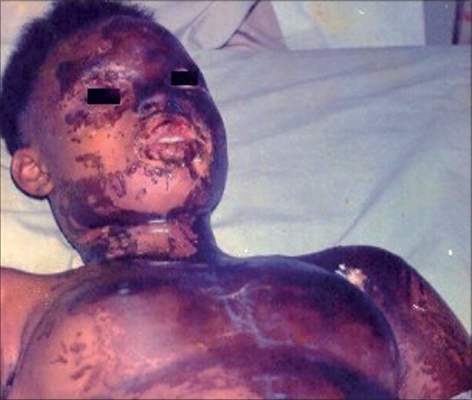
Patient at presentation 36 hours after chemical assault

**Figure 2:**
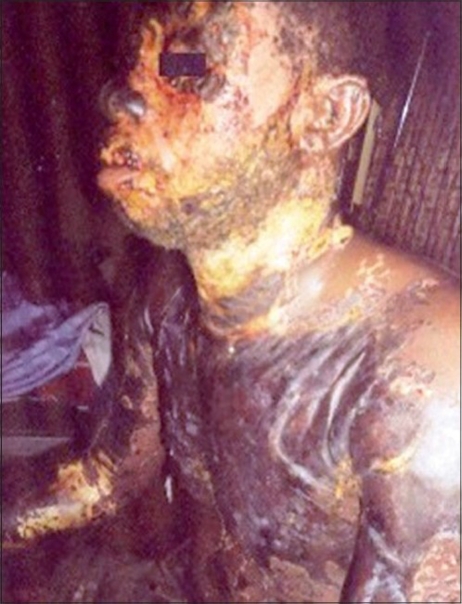
A patient with chemical burns with onset of eschar separation

**Figure 3:**
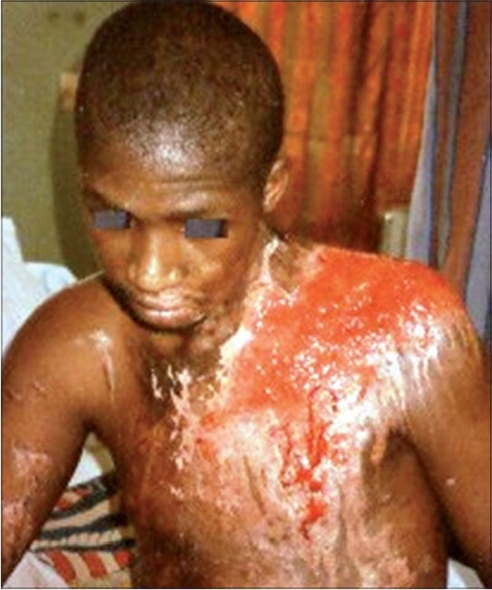
Full-thickness wound following eschar separation in a patient with chemical burns

Injuries were usually sustained while patients were alone and the chemicals were commonly kept in a plastic basin or cellophane bags. The chemical was either poured on the face of the patient during an attack or while the patient was sleeping in one of the cases. The time before presentation ranged between 24 hours to three months with an average of ten days. The depth of the burns was usually not difficult to judge as all the patients came after 24 hours [Figure 1]. They therefore presented with thick, hard, darkened skin or full-thickness wounds when the eschar was already separating [Figures 2-3]. The area of the burns ranged between 8- 70% of the total body surface with a mean of 30.2%.

The face was the most commonly affected with 21 cases (75%) followed by 18 (22.5%) cases with neck injuries while the least affected area was the posterior trunk (two, 0.7%). Raw eggs, palm oil, gentian violet and engine oil were the substances applied immediately after the injuries.

Complications observed included septicemia, respiratory distress, blindness, renal failure, mentosternal contractures, ectropion, axillary contractures, hypertrophic scars, keloids and skin depigmentation. The average period spent in the hospital was 126.5 days with a range of 15-216 days.

Three of the patients died bringing the mortality to 10.7%.

## DISCUSSION

The current review of chemical burns is unique in that it deals with chemical burns sustained from places other than laboratories and industries already associated with extensive burn injuries. In the North America[1,2] and Europe,[3,4] acid burns are generally regarded as uncommon industrial accidents and they are rarely associated with assault.[5,6] In some parts of the developing world however, acid assaults constitute a significant burden of burn injuries and they appear to follow different patterns in different regions.[7]

Most (74.1%) of our patients were assaulted by jilted lovers, business associates or family members as a result of squabbles while 7.8% of them were attacked by armed robbers who were usually still at large at the time of presentation. The agents used could therefore not be ascertained in most cases. In Bangladesh[8] and China,[9] most acid assault victims are young women who had refused a particular suitor while in Jamaica,[10] the victim is most often an unfaithful husband.

A previous review conducted on burn injuries about a decade ago in this center[11] showed only 8/242 (3.3%) patients with chemical burns over a three year period compared to 28/485 (5.8%) patients in the five year study discussed in the current report. There is therefore an upsurge in the cases of chemical injuries. The average burnt surfaces in the previous and the current studies were 19 and 30.2% respectively. Delay in presentation was also observed along with poor knowledge of first aid.

Although, chemical burns were found mostly among young adults in this study, some middle-aged patients as well as a two-year old child were also involved with the youngest of our patients being a 2 year old boy.

Brandy *et al*.[10] reported that many Jamaicans learned the value of carrying household chemicals such as sulphuric acid from batteries or sodium hydroxide obtained from cleaning supplies. These are used as a means of defence among the lower socioeconomic groups who could not afford handguns. They submit that dangerous chemicals used as defensive weapons have extended to the use of chemicals for assault. While chemicals are not commonly used as a means of defence in Nigeria, the fact that they can be easily obtained either from rubber plantations or from “battery water” from artisans, make the use possible for assailants. Sulphuric acid is the most common chemical from these two sources besides NaOH (sodium hydroxide) which is used locally in making soap for domestic use.

Materials used as first aid included raw eggs, palm oil and gentian violet. The chemical agents used to cause the burn injuries were not known in our patients except in two patients who attempted suicide as the assailants were usually at large at presentation. This is contrary to many other reported cases where the specific agents were stated.[1,2]

Bromberg *et al*.[12] had noted that tissue damage continues to increase as long as the chemical is in contact with the tissue and hence, treatment is necessary to remove the causative substances as quickly as possible by washing with large volumes of water. Washing is presumed to cause dilution and elimination of chemical substances, alteration of the chemical reaction, suppression of any raised tissue metabolism, to have an anti-inflammatory action and to return skin pH levels to normal.

Yano *et al*. opined that washing should be continued until the skin pH returns to normal and should begin as soon as possible after the injury.[13] Unfortunately, our patients used many other substances apart from water thereby sustaining extensive and deep burns. This may also explain why all our patients had to be admitted apart from the fact that the injuries were extensive.

Only five patients had burn surface areas of ≤10% while the average total burn surface area was 30.2%. There was no case of industrial accident in the current review. Most burns from chemicals as observed in a burns center in Portugal were minor and involved the hands, lower limbs and trunk[14] as they were accidental.

Our series shows that the majority of the injury cases (921, 75%) were from assault which occurred in varying places like workshops, along the road and at home. In contrast, the Portuguese report [14] shows most of the chemical burn injuries to be accidental and occurring in the workplace.

**Figure 4:**
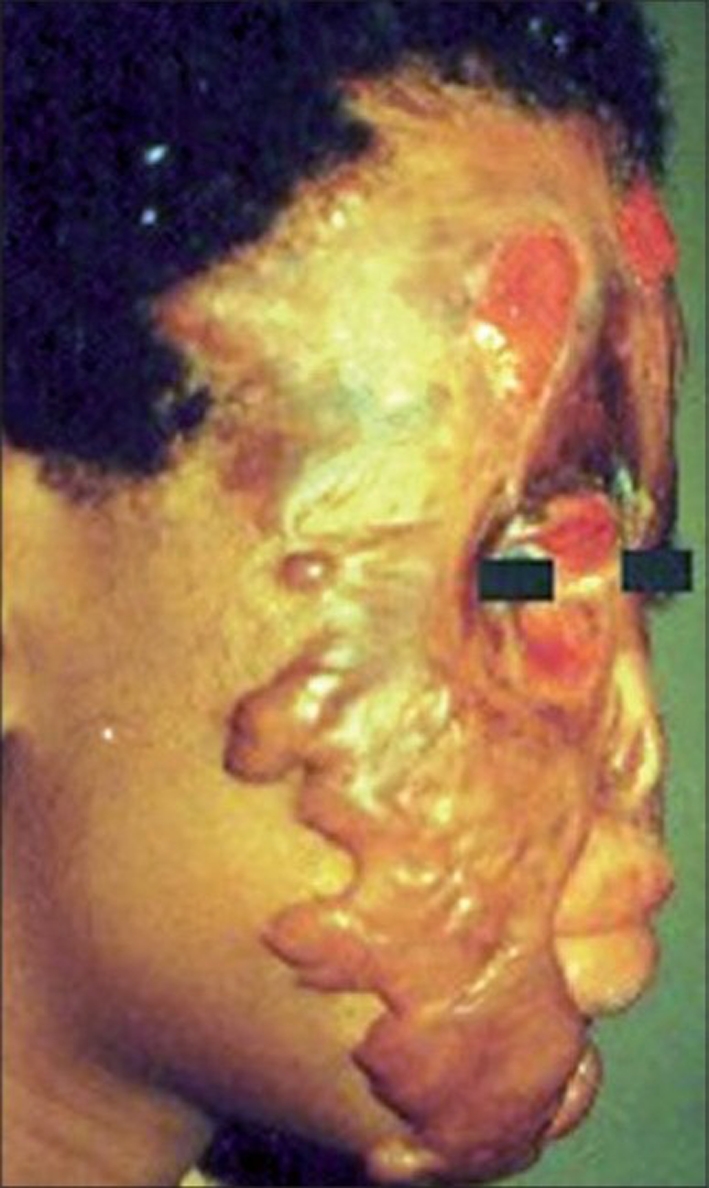
Patient with chemical burn complications of hypertrophic scar and ectropion. Note the remaining wound on the forehead

Most of our patients presented with severe septic burns or burn complications like contractures, ectropion and severe facial deformities [Figures 1-4].

In cases when the patients presented early, a number of them could not afford the required early excision and blood transfusion. They then had to wait till the eschar separated and the wound grafted. This was responsible for the prolonged hospital stay of these patients. Many were discharged with remnant deformities meant to be taken care of when more funds were available. Only two of the patients had early excision and grafting of the wounds with fewer complications and reduced hospital stay.

Two of our patients had cornea perforation in one of their eyes, one had bilateral corneal perforation while ten had severe ectropion, which was released and grafted. Bromberg *et al*.[12] had earlier noted that serious ocular damage is a common sequel of corrosive injury whereas it is rare following thermal injury.

In conclusion, chemical burn from assault is becoming common in Nigeria. The agent is usually unknown as the assailants are usually at large at presentation. Education to let the people know the implication of these injuries and its consequences on the victims is important in its prevention. People should also be warned of the potential hazards of keeping chemicals at home where any angry youth can readily use them for vengeance. Stiff penalty for the attackers will help in curbing this problem and preventing the economic loss which occurs as a result of prolonged hospital stay and the attending psychological problems as young adults.

Education of the populace on the first aid measures following chemical burns is also important. Rather than using just any substance, they should quickly irrigate the site(s) of chemical injuries with copious amounts of water.

Efforts should also be made to prevent easy access to chemicals by the people who have no business with them.

These will reduce the use of this barbaric means of settling disputes and the usual complications that arise from the injuries.

Treatment of burns especially from assaults as observed in these cases should be made free of charge to allow prompt treatment and prevent avoidable complications by early surgery.

## References

[1] Leonard LG, Scheulen JJ, Munster AM (1982). Chemical burns: Effect of prompt first aid. J Trauma.

[2] Skyes RA, Mani MM, Hiebert JM (1986). Chemical burns: Retrospective review. J Burn Care Rehabil.

[3] Tremel H, Brunier A, Weilemann LS (1991). Chemical burns caused by hydrofluoric acid: incidence, frequency and current status of therapy. Med Klin.

[4] Munoch DA, Daray CM, Whallet EJ, Dickson WA (2000). Work related burns in South Wales 1995-1996. Burns.

[5] Brozka W, Thornhill HL, Haward S (1985). Burns: Causes and risk factors. Arch Phys Med Rehabil.

[6] Krob MJ, Johnson A, Jordan MH (1986). Burned and battered adults. J Burn Care Rehabil.

[7] Purdue CF, Hunt JL (1990). Adult assault as a mechanism of burn injury. Arch Surg.

[8] Faga A, Scevola D, Mezzetti MG, Scevola S (2000). Sulphuric acid burn women in Bangladesh: A social and medical problem. Burns.

[9] Yeong EK, Chen MT, Mann R, Lin TW, Engrav LH (1997). Facial mutilation after an assault with chemicals: 15 cases and literature review. J Burn Care Rehabil.

[10] Brandy J, Arscott GD, Smoot EC, Williams GD, Fletcher PR (1996). Chemical burns as assault injuries in Jamaica. Burns.

[11] Achebe UJ, Akpuaka FC (1989). Chemical burns in Enugu. West Afr J Med.

[12] Bromberg BE, Song IC, Walden RH (1965). Hydrotherapy of chemical burns. Plast Reconstr Surg.

[13] Yano Y, Hata K, Matsuka O, Ito O, Matsuda H (1993). Experimental study on alkaline skin injuries-periodic changes in subcutaneous tissue Ph and the effect exerted by washing. Burns.

[14] Da Silva JC, Bento C, Coelho MJ, Almeida MA (2002). Chemical burns: A clinical report on 30 cases. Ann Burns Fire Dis.

